# Artificial intelligence-based prognostic modeling in temporomandibular disorders and chronic orofacial pain: a critical conceptual review

**DOI:** 10.22514/jofph.2026.049

**Published:** 2026-07-12

**Authors:** Mohammad H. Al-Harthy

**Affiliations:** ^1^Department of Basic and Clinical Oral Sciences, Faculty of Dental Medicine, Umm Al-Qura University, 24231 Makkah, Saudi Arabia

**Keywords:** Temporomandibular disorders, Chronic orofacial pain, Artificial intelligence, Machine learning, Prognostic modeling, Biopsychosocial model

## Abstract

This conceptual review (ⅰ) analyzes the outcomes predicted, data modalities, modeling approaches, validation strategies, and reporting quality of existing artificial intelligence (AI)-driven prognostic models in temporomandibular disorders (TMD) and chronic orofacial pain (OFP); (ⅱ) identifies enduring methodological and ethical constraints that hinder clinical translation; and (ⅲ) proposes a pragmatic research framework to guide the responsible development of clinically relevant prognostic tools for TMD and OFP. The review covers peer-reviewed and other relevant publications from the previous decade, emphasizing AI or machine-learning (ML) based models for prognosis, outcome prediction, or trajectory modeling in TMD and OFP populations. Established paradigms, including the Transparent Reporting of a multivariable prediction model for individual Prognosis Or Diagnosis plus Artificial Intelligence extension (TRIPOD + AI) and the Prediction model Risk Of Bias Assessment Tool (PROBAST), were used to assess the literature. Methodologies remain highly inconsistent, and current literature lacks the volume and rigor required for clinical translation. Most AI research has concentrated on diagnostic classification rather than prognostic modeling. Small sample sizes, short follow-up, single-center datasets, omission of psychosocial factors, and a general lack of external validation hamper the few prognostic studies that exist. Most model outputs are neither clinically actionable nor suitable for direct use in treatment decisions, limiting their value for clinicians and their potential impact on patient outcomes. Research applying AI to forecast TMD and OFP remains in its early stages. Without a prognosis-first research design, longitudinal data integration, inclusion of biopsychosocial predictors, and clinically significant outcome objectives, existing models are unlikely to influence clinical practice. Clear research objectives, reporting criteria, and ethical norms must be established before AI-based prognostic models can be confidently adopted in TMD and OFP clinical practice. Future objectives comprise establishing multicenter longitudinal cohorts, conducting trajectory-based modeling, employing federated learning for external validation, and initiating prospective clinical trials to demonstrate clear clinical benefit.

## 1. Introduction

Temporomandibular disorders (TMD) and orofacial pain (OFP) encompass a heterogeneous group of musculoskeletal and pain-related conditions that affect the masticatory system, temporomandibular joints (TMJs), and associated structures [1, 2]. These conditions constitute a substantial global health concern, with a recent meta-analysis estimating a symptom-based prevalence of approximately 30–40% and an annual incidence of clinically verified first-onset TMD of nearly 4% [3, 4]. A substantial subset of affected individuals develops persistent pain, functional limitations, and psychosocial distress, with projections indicating that global prevalence may reach nearly 44% by 2050 [3]. The introduction of standardized diagnostic criteria for TMD (DC/TMD) has improved the reliability and consistency of diagnosis [1, 2]; however, diagnostic classification alone has proven insufficient for guiding personalized treatment strategies or predicting long-term outcomes [1, 5]. Consequently, clinicians managing these patients encounter substantial prognostic uncertainty. Questions such as “Which patient is at risk of developing chronic pain?”, “Who is likely to respond to conservative treatment?”, and “When should a more intensive approach be considered?” remain largely unanswered by current clinical resources [6, 7]. Although some longitudinal investigations, most notably the Orofacial Pain Prospective Evaluation and Risk Assessment (OPPERA) studies, have identified risk factors and clinical characteristics that may inform the onset or progression of TMD, these studies have yet to yield concrete, validated diagnostic or prognostic tools suitable for individual-level clinical decision-making [5, 8]. The multifactorial complexity of TMD, driven by numerous interacting biological, behavioral, and psychosocial factors, further underscores the challenge of translating population-level risk data into actionable clinical predictions [5].

Beyond the identification of individual risk factors, prognosis in TMD/OFP increasingly requires consideration of the dynamic, multidimensional interactions among biological, psychological, behavioral, and social domains, as demonstrated in contemporary biopsychosocial models and longitudinal cohort research [9, 10, 11]. Longitudinal evidence has shown that TMD/OFP are characterized by nonlinear trajectories, symptom fluctuations, and heterogeneous treatment responses, rather than predictable linear courses [11, 12]. Therefore, traditional single-variable prognostic models and clinician intuition alone are often insufficient for accurate individual-level prediction [13, 14].

The majority of existing TMD/OFP prognostic studies have relied on regression models, including logistic and linear regression, to examine the relationships between baseline predictors and future pain and function at the population level [5, 15, 16]. While these models provide valuable information at the group level, they are often poorly calibrated, meaning that predicted probabilities do not closely correspond to observed outcome frequencies. This discrepancy may result in overconfident or underconfident estimates and generally yield only moderate performance for individual-level prediction of future pain and function at a single time point [13, 17]. Furthermore, many studies focus on a single variable or domain, such as psychological distress or pain intensity, while neglecting other variables that may provide unique information from multiple data domains and could be critical to the pathways underlying TMD/OFP chronicity or recovery. This limitation constitutes a key motivation for employing machine learning (ML) models, which have demonstrated improved predictive performance by capturing interactions across multiple domains of data [18, 19].

Artificial intelligence (AI), particularly ML, offers distinctive analytical capabilities, including the ability to model high-dimensional, nonlinear relationships across large numbers of variables simultaneously [20, 21]. Interpretable ML approaches have recently been applied to TMD risk modeling in adults [20], and ensemble learners utilizing routine clinical parameters such as pain intensity, mouth opening, and pressure pain thresholds have been shown to predict TMD outcomes [21].

In medical fields including oncology, cardiology, and neurology, AI-based prognostic models have demonstrated promising performance and, in several applications, measurable improvements over traditional approaches [19, 22]. Broader frameworks for AI in pain medicine have similarly outlined opportunities for implementation as well as remaining requirements for successful translation into clinical practice [23]. Whether comparable gains are achievable in TMD and OFP remains an important open question [23].

In the fields of dentistry and OFP research, AI applications have primarily focused on diagnostic functions, particularly imaging-based detection and automated classification of clinical conditions [24, 25]. Systematic reviews indicate that the majority of AI studies in TMD/OFP have concentrated on diagnostic classification, whereas prognostic AI models designed to forecast future pain trajectories, assess the risk of chronicity, or predict treatment response have received comparatively limited attention [26, 27]. This diagnostic-prognostic disparity reflects a broader trend observed in health AI research [19, 22].

The limited number of AI-based prognostic studies in TMD/OFP demonstrates considerable methodological heterogeneity. Variations exist across multiple domains, including clinical, imaging, self-report, or mixed modalities; outcome definitions; feature selection strategies; model types; and validation procedures [26, 27]. In addition, many studies lack transparency in reporting key methodological details, such as approaches to missing data, internal or external validation processes, and assessments of model calibration [28, 29]. These deficiencies hinder the interpretation, reproducibility, and synthesis of findings in prediction model research [28]. From a clinical perspective, robust AI-based prognostic models could directly support several key decision points in TMD/OFP management, including the early identification of patients at high risk of developing chronic pain, stratification of individuals according to anticipated outcomes, and personalized management planning informed by predicted treatment benefit [17, 30]. Such capabilities could help address a long-standing gap in OFP care, where clinicians often rely on trial-and-error approaches in the absence of reliable prognostic tools. The substantial burden of chronic pain, which affects an estimated 20% of adults [31], underscores the urgency of developing these tools. Recent reviews on AI in pain management have highlighted a shift from diagnostic classifiers toward integrated prognostic and decision-support tools [32].

Despite the growing interest in AI-based prognostic models, significant concerns persist regarding bias, generalizability, interpretability, and practical clinical utility [33]. Models developed using data from narrowly defined cohorts or single-institution datasets may perform suboptimally when applied to heterogeneous patient populations or diverse healthcare settings [28, 34]. Moreover, algorithmic bias can arise even in the absence of explicit consideration of protected attributes, potentially resulting in disparities in performance across different patient subgroups [35]. Algorithms lacking transparent reasoning may face resistance from clinicians and impede collaborative decision-making and accountability, thereby limiting adoption in clinical practice and constraining their potential benefits for patient care [33]. Collectively, these considerations underscore the need for a focused evaluation of current AI research to assess the effectiveness of prognostic models in TMD/OFP beyond diagnostic applications [26, 27, 36, 37]. Such an assessment should systematically examine existing models, the data modalities employed, outcome definitions, validation approaches, and reporting practices, in alignment with established standards for prognostic research [30, 38]. Without this scrutiny, premature deployment of AI-driven prognostic models could lead to misapplication and potential harm to both patients and healthcare systems [19, 39].

In this context, the present review aims to evaluate the current status of AI-based prognostic models within the TMD/OFP field [37, 40]. Specifically, it seeks to (ⅰ) analyze the outcomes predicted, data modalities used, modeling approaches adopted, validation strategies employed, and reporting quality of existing studies; (ⅱ) identify enduring methodological and ethical constraints that limit clinical translation; and (ⅲ) propose a pragmatic framework to support the responsible development of clinically relevant prognostic tools for TMD/OFP [34, 38].

This review is intended to clarify the current state of AI research in TMD and OFP, highlight key gaps, and reorient future efforts toward the development of clinically meaningful prognostic tools that address important unmet clinical needs.

## 2. Review approach and methodology

The present review offers a conceptual analysis, integrating and interpreting the available evidence on AI-driven prognostic modeling in TMD/OFP. Conceptual reviews are designed to illuminate theoretical frameworks, identify knowledge gaps, and propose research avenues, rather than to provide a comprehensive inventory of all published literature [40]. Established reporting standards, including the Transparent Reporting of a Multivariable Prediction Model for Individual Prognosis or Diagnosis + Artificial Intelligence (TRIPOD + AI) statement, now guide the evaluation of AI-based prediction models, with recent extensions addressing large language models (TRIPOD-LLM) [41]. This approach is especially appropriate when the existing evidence is diverse or methodologically immature, as is the case in this domain [42].

The original search was conducted in PubMed/MEDLINE and Scopus [43, 44]; however, institutional access to Scopus was no longer available during manuscript revision. The search was therefore updated using PubMed/MEDLINE, Web of Science Core Collection, IEEE Xplore, and Google Scholar, with the top 100 results per query used as a supplementary cross-check. A three-concept search framework—condition (TMD/orofacial pain) AND AI/ML methodology, AND prognostic focus—was applied to all databases, with search syntax adapted to each database’s indexing conventions [44]. The search strategy incorporated terminology pertinent to TMD/OFP, including “temporomandibular disorders”, “orofacial pain”, and “craniomandibular disorders”; AI or ML, including “machine learning”, “deep learning”, “artificial intelligence”, and “predictive algorithms”; and prognosis or outcome prediction, including “prognosis”, “outcome prediction”, “risk stratification”, and “treatment response”. The search covered 2015–2025, was limited to English-language human studies, and was supplemented by citation chasing. The three indexed databases yielded 301 records (PubMed: 94; Web of Science: 167; IEEE Xplore: 40). After restricting the records to studies with a definitive prognostic aim, only 11 studies were identified, underscoring the diagnostic–prognostic disparity examined throughout this review. The final narrative synthesis was based on these prognostic studies, as well as the wider AI/ML literature in TMD/OFP, key works in prognostic methodology, reporting frameworks, and other references identified through citation chasing. A total of 77 publications were included. Selection was based on conceptual relevance and methodological rigor rather than strict inclusion and exclusion criteria, consistent with the conceptual nature of this review. The loss of access to Scopus is acknowledged as a limitation. Because Scopus offers broader engineering and interdisciplinary indexing than PubMed, its absence may have reduced the yield of citation chasing for engineering-focused ML studies relevant to OFP. However, this risk was mitigated by the inclusion of IEEE Xplore, which directly indexes core engineering and computer-science venues likely to publish such work, and by Web of Science, whose Science Citation Index Expanded provides substantial overlap with Scopus coverage. Google Scholar further served as a supplementary cross-check, capturing preprints and conference proceedings not indexed in traditional databases. The included literature was evaluated using criteria derived from established prognostic research frameworks and reporting standards, including the TRIPOD + AI statement [29], the Prediction model Risk of Bias Assessment Tool (PROBAST) [28], and the prognosis research strategy (PROGRESS) [30, 45].

For each eligible prognostic study, the synthesis considered the predicted outcome, follow-up design, data modality, predictor domains, modeling approach, validation strategy, reporting completeness, generalizability, and potential clinical applicability. Findings were then synthesized narratively and organized according to outcome domains, data modalities, modeling methodologies, and translational readiness. A narrative synthesis approach was considered appropriate because methodological heterogeneity across studies precluded meaningful quantitative aggregation [42]. This interpretation is consistent with a recent systematic review of AI applied to TMJ magnetic resonance imaging (MRI), which showed that deep-learning approaches are increasingly effective for segmenting TMJ structures and classifying disc position, whereas prognostic endpoints remain largely unaddressed [46].

## 3. Conceptual framework for AI-based prognostic modeling

### 3.1 Prognosis versus diagnosis: a fundamental distinction

Diagnostic classification aims to identify the presence or subtype of a disease at a given time point; prognosis, by contrast, seeks to estimate future outcomes, including pain persistence, functional impairment, treatment response, and risk of chronicity [13]. In TMD and OFP, diagnosis alone rarely dictates management decisions. Patients sharing the same DC/TMD diagnosis can follow markedly different clinical trajectories, driven by individual variation in psychosocial factors (*e.g.*, anxiety, catastrophizing, coping style), biological predisposition, pain processing mechanisms, and behavioral and environmental factors. This variability underscores why diagnosis-centric models are insufficient for guiding individualized care [12, 47].

Currently, AI applications in dentistry are primarily based on static or two-dimensional cross-sectional images and focus largely on diagnostic performance [24, 25]. Performance is commonly measured using metrics such as accuracy, precision, F1-score (a composite metric that balances precision and recall), or the area under the receiver operating characteristic curve (AUC-ROC), which quantifies a model’s ability to discriminate between outcome groups [24, 25]. However, these metrics do not directly address the most clinically relevant questions for patients, such as “Will my current pain become chronic?” or “Will this treatment plan be effective?” Addressing such questions requires dynamic information, including temporal patterns and trends, and modeling changes in patient state over time [13].

### 3.2 Core data domains for AI-based prognostic modeling in TMD/OFP

An effective AI-based prognostic framework for TMD/OFP must integrate multiple data domains that collectively reflect both disease mechanisms and patient experience. Four core domains are particularly relevant (Table 1, Ref. [7, 9, 10, 16, 47, 48, 49]): (1) clinical and demographic data, (2) psychosocial and behavioral factors, (3) imaging and structural data, and (4) patient-reported outcome measures (PROMs). Each domain provides complementary information that, within a multidimensional framework, enables prognostic modeling capabilities unlikely to be achieved using a single-domain approach. The utilization of these data domains in existing studies is discussed in detail in Sections 4 and 5.

**Table 1.** Core data domains for AI-based prognostic modeling in TMD/OFP.

Domain	Key Variables	Prognostic Relevance	Current Integration in AI Models
Clinical and demographic	Age, sex, pain intensity, pain duration, symptom fluctuation, jaw function, prior treatment history	Foundational prognostic inputs; demonstrated predictive value for incident TMD in OPPERA cohort studies [48, 49]	Commonly included but often without psychosocial co-variables; nonlinear interactions are rarely modeled
Psychosocial and behavioral	Pain catastrophizing, anxiety, depression, sleep disturbance, coping behaviors, and other manifestations of psychological distress	Among the strongest predictors of pain persistence and disability in TMD and chronic pain populations [9, 10, 16]	Frequently underrepresented despite well-established prognostic importance; data availability biases favor imaging
Structural and imaging	TMJ condylar morphology, disc displacement (DC/TMD disc displacement categories), osteoarthritic (DC/TMD: degenerative joint disease) changes, MRI findings	May contribute in selected subgroups, but correlations with symptom severity or chronicity are weak and inconsistent in isolation [7, 47]	Most technically mature area; high diagnostic accuracy but limited prognostic value when isolated from other domains
Patient-reported outcome measures (PROMs)	Pain intensity (NRS/VAS), jaw functional limitation, quality-of-life indices (OHIP, SF-36)	Capture disease burden and longitudinal symptom trajectories; essential for tracking symptom evolution [10]	Rarely incorporated as repeated measures; typically captured at a single baseline time point

*PROMs: patient-reported outcome measures; TMJ: temporomandibular joint; DC/TMD: Diagnostic Criteria for Temporomandibular Disorders; MRI: magnetic resonance imaging; NRS: numeric rating scale; VAS: visual analog scale; OHIP: Oral Health Impact Profile; SF-36: Short Form Health Survey-36; TMD: temporomandibular disorders; OPPERA: Orofacial Pain Prospective Evaluation and Risk Assessment; AI: artificial intelligence.*

### 3.3 Temporal dynamics and the need for longitudinal modeling

Unlike diagnostic models, prognostic AI systems must account for time. Pain trajectories in TMD and OFP can evolve, remit, or episodically flare in patterns that are potentially influenced by psychosocial factors and therapeutic interventions [10, 16]. The OPPERA studies demonstrated that biopsychosocial characteristics shift over time and that these longitudinal changes are meaningfully associated with TMD outcomes [10]. Approaches such as survival analysis-augmented ML [50], or deep learning architectures such as recurrent neural networks (RNNs), which are designed to learn from sequential data and capture temporal dependencies [51], are therefore conceptually better suited to prognostic tasks than static cross-sectional classifiers. Practically, RNNs and their variants, including long short-term memory (LSTM) networks [51], are designed to process patient data collected across multiple visits, such as repeated pain scores, functional assessments, and psychosocial measures, sequentially. This allows the model to learn how a patient’s current status relates to prior symptom history, making it well-suited for detecting trends such as remission–relapse cycles or slow deterioration that could be missed by a single time-point assessment. In a complementary approach, survival analysis-augmented ML integrates time-to-event analysis with ML, predicting not only whether a clinical outcome (*e.g.*, transition to chronic pain) is likely, but also when it may occur, while capturing complex nonlinear relationships among risk factors. These techniques align with the clinical reality of TMD/OFP, in which patients often follow irregular trajectories of improvement, plateauing, and relapse rather than a predictable linear course.

Most AI studies in TMD and OFP are based on cross-sectional datasets, which are not consistent with the forward-looking nature of clinical prognosis. These studies train models to classify the current state rather than predict future outcomes, a distinction that is often overlooked in study descriptions but is critical for the validity of prognostic claims.

### 3.4 Explainability and clinical trust

For prognostic AI to inform clinical decision-making, clinicians must understand why a model predicts poor prognosis or treatment resistance, particularly in pain medicine, where shared decision-making is essential [52]. Explainable AI techniques are therefore highly relevant. Local interpretable model-agnostic explanations (LIME) approximate complex model behavior locally, providing explanations for individual predictions in simple, interpretable terms [53]. SHapley Additive exPlanations (SHAP) assign each input variable a contribution score based on cooperative game theory, quantifying its influence on the prediction [54]. These methods, together with feature importance analysis and other model-agnostic interpretability tools, are integral to clinical translation. Models operating as “black boxes” may achieve strong statistical performance but fail to gain clinical acceptance, particularly in conditions influenced by both psychosocial and biomechanical factors [52, 55]. The integrated biopsychosocial AI prognostic framework proposed in this review, encompassing the data domains, temporal integration, modeling, and clinical output stages described in Sections 3.1–3.4, is illustrated in Fig. 1.

**Fig. 1.** Integrated biopsychosocial AI prognostic framework for TMD/OFP. The framework presents a four-stage prognostic pipeline: (Stage 1) multimodal data collection across four biopsychosocial domains, namely clinical/demographic, psychosocial/behavioral, structural/imaging, and patient-reported outcome measures; (Stage 2) temporal integration, including longitudinal linkage, trajectory modeling, and data leakage prevention safeguards; (Stage 3) AI/ML model development with explainability and validation, including calibration assessment; and (Stage 4) clinical outputs, including individualized risk stratification, modifiable risk driver identification, management pathway linkage, and shared decision-making. Cross-cutting requirements include TRIPOD + AI, PROBAST, calibration assessment, fairness metrics, and stakeholder engagement. (Figure constructed based on the frameworks and evidence discussed in this review using Microsoft® PowerPoint for Mac, V. 16.107). TMD: temporomandibular disorders; DC/TMD: Diagnostic Criteria for Temporomandibular Disorders; PHQ-9: Patient Health Questionnaire-9; GAD-7: Generalized Anxiety Disorder-7; TMJ: temporomandibular joint; MRI: magnetic resonance imaging; CBCT: cone-beam computed tomography; OHIP: Oral Health Impact Profile; NRS: numeric rating scale; VAS: visual analog scale; QoL: quality of life; AI: artificial intelligence; ML: machine learning; RF: random forest; SHAP: SHapley Additive exPlanations; LIME: Local Interpretable Model-Agnostic Explanations; TRIPOD: Transparent Reporting of a Multivariable Prediction Model for Individual Prognosis or Diagnosis; PROBAST: Prediction Model Risk of Bias Assessment Tool.

## 4. Recent research on AI-based prognostic models in chronic OFP and TMD

### 4.1 Imbalance in diagnostic-prognostic ratios

Although interest in AI is increasing in pain management and dentistry, the evidence base for AI-based prognostic modeling in TMD and chronic OFP remains sparse and is markedly skewed toward diagnostic purposes. Most published AI research employs supervised learning to categorize imaging findings, disease presence, or diagnostic categories at a single time point [24, 26, 27]. Although these studies frequently report high levels of accuracy, their predictive value for clinically relevant outcomes, including pain persistence, progression of functional impairment, and treatment nonresponse, is usually not evaluated [13, 30].

Recent systematic reviews corroborate this diagnostic-prognostic disparity. Jha *et al*. [26] (2022) found that, across several deep learning models used for the diagnosis of TMD, the reported classification accuracies of AI algorithms ranged from 84% to 99.9%; however, all studies were judged to have a consistently high risk of bias, and the certainty of evidence was therefore rated as very low [26]. Farook *et al*. [27] (2021) similarly reported that ML solutions in oral and OFP management primarily focus on diagnostic tasks instead of outcome prediction, highlighting a significant gap in the development of prognostic tools for patient care and treatment planning in these fields. Both reviews indicate that the literature largely lacks actual prognostic modeling characterized by temporal validation, longitudinal outcomes, and clinically interpretable endpoints [26, 27]. This distinction is important, as many studies described as “predictive” in the TMD AI literature do not fulfill established criteria for prognostic research and are more accurately classified as diagnostic AI [17, 38, 56].

To summarize the landscape of existing research in relation to the first objective of this review: the types of outcomes predicted have largely been limited to diagnostic classification rather than true prognostic endpoints; the data modalities utilized have been predominantly imaging-based or limited to single-domain clinical variables; and the modeling approaches adopted have mainly relied on supervised learning classifiers applied to cross-sectional data.

### 4.2 Data from other chronic pain areas

Given the limited number of prognostic AI studies specific to TMD/OFP, evidence from related chronic pain fields may offer useful reference points; however, such insights must be interpreted with careful consideration of the clinical context.

In examining ML techniques in pain research, Lötsch and Ultsch (2018) demonstrated the potential of ML techniques to detect complex patterns in clinical data and to predict pain phenotypes [57]. Parallel analyses in low back pain, conducted by Tagliaferri *et al*. [58] (2020), found no studies employing AI specifically to predict low back pain prognosis, a gap that directly parallels the situation in TMD/OFP. The evidence reviewed suggests that shared prognostic factors, including psychological components, baseline interference, and pain history, are relevant across TMD and other chronic pain populations [10, 16]. Nevertheless, the presence of shared factors does not guarantee direct transferability. TMD/OFP involves jaw-specific anatomical, behavioral, and psychological elements, including jaw function, parafunctional habits, and associated anxiety and sleep disturbances, that necessitate domain-specific prognostic models [13, 52]. A systematic review and meta-analysis of deep-learning models for TMJ arthropathies further demonstrated high diagnostic sensitivity and specificity, however, the field remains primarily diagnostic rather than prognostic in orientation [59].

### 4.3 Growing prognostic indicators from studies on TMD

Although a dedicated focus on AI-based prognosis has been lacking (Table 2, Ref. [16, 20, 21, 26, 27, 48, 57, 60, 61, 62, 63]), a few studies have begun to examine prognostic factors within the context of TMD. Most prognostically relevant data have been derived from the OPPERA study. In a prospective cohort of 3263 participants, Fillingim *et al*. [16] (2013) identified several premorbid psychological characteristics, notably the severity of somatic symptoms, as predictors of first-onset TMD. Bair *et al*. [48] (2013), using lasso regression and random forests to analyze 202 baseline risk factors from the OPPERA study, found that the most important predictors of incident TMD were comorbid pain, psychosocial parameters, and baseline pain sensitivity [48]. Supervised cluster analysis was used by Bair *et al*. [60] (2016) to develop a classification system that identified three biopsychosocial subgroups, designated as the adaptive, pain-sensitive, and global symptoms clusters. These clusters were significantly predictive of both the prevalence and incidence of TMD [60]. More recently, Al Turkestani *et al*. [61] (2024) developed a comprehensive patient-specific prediction model for TMJ osteoarthritis (DC/TMD: defined degenerative joint disease) progression using an ensemble framework that integrated clinical, imaging, and biological variables with clearly defined longitudinal outcomes over a 2–3-year follow-up, further illustrating the feasibility of a prognostic-first AI approach in TMD [61].

**Table 2.** Summary of key AI/ML-related prognostic studies in TMD and OFP.

Study	Population/Sample	AI/ML Method	Key Prognostic Finding	Limitations	PROBAST Risk of Bias	Calibration Reported	PROBAST Analysis Domain Concerns
Lee *et al*. [62] (2025)	239 TMD patients (161 women, 78 men); acute (<6 months) *vs.* chronic (≥6 months); single center	Logistic regression and deep neural network (MLP) with SHAP interpretability analysis	Bruxism, VAS, sleep problems, TMJ noise, ADD (encompassing DC/TMD disc displacement categories), and joint space narrowing were identified as significant predictors of chronic TMD; DNN AUC-ROC 0.7949 *vs.* logistic regression 0.7550	Retrospective single-center design; modest sample size for DNN; no external validation; self-reported behavioral data	High (retrospective, single-center, no external validation, small sample for DNN)	No (AUC-ROC only)	Missing data handling not reported; no penalization or shrinkage; no calibration—AUC-ROC only
Xu *et al*. [63] (2026)	584 TMD patients (755 TMJ MRI datasets); retrospective (2022–2024)	ML algorithms with SHAP interpretation; MRI features (disc position, morphology, signal, perforation, joint effusion, condylar movement, bony changes, lateral pterygoid muscle)	LightGBM was the best predictive performer (AUC 0.899); SHAP identified age, disc position, and condylar movement as the top three contributing features; top 9 SHAP-ranked features achieved highest diagnostic performance (AUC 0.829)	Retrospective single-center design; imaging-only features without clinical or psychosocial variables	High (retrospective, imaging-only, no psychosocial integration)	Calibration curves were plotted to assess discrimination and model calibration	No external validation; single random train/test split without repeated cross-validation; calibration curves reported but no calibration-in-the-large or calibration slope statistics; no missing data handling strategy described; VAS-based pain classification threshold not externally justified
Fillingim *et al*. [16] (2013)	OPPERA cohort; n = 3263 community controls	Traditional regression (prospective cohort design)	Premorbid somatic symptoms and psychological distress predict first-onset TMD	Not framed as AI; no ML algorithm applied; single-center enrollment	Moderate (prospective cohort design, but not framed as AI/ML; single-center enrollment)	No	Standard regression without shrinkage; no calibration reported
Cui *et al*. [20] (2024)	949 adults (799 development + 150 external test cohort); Stomatology Hospital of Jilin University	Five ML algorithms (RF, XGBoost, LR, DT, GBDT) with SHAP interpretation; RF with 7 features selected as the final model	RF model achieved AUC 0.892 (training), 0.854 (internal validation), 0.857 (external test); top predictors: anxiety, malocclusion, unilateral chewing, clenching teeth, gender	Single-center data; external validation from the same hospital (different time period); self-reported behavioral variables	High (single-center development; external validation from the same institution at a different time period)	No (AUC-ROC only)	Missing data handling not described; no calibration curve; over-optimism risk given single-center design
Yıldız *et al*. [21] (2024)	125 TMD patients + 103 matched controls; cross-sectional; clinical measurements (VAS, MMO, PPT, CROM, OBC, HADS, OHIP-14)	Over 20 ML algorithms compared; Bagging ensemble with MARS as base learner	Bagging (MARS) achieved an accuracy of 0.8966, AUC 0.9387; top 5 predictors: pain intensity, MMO, TMJ lateral movement, PPT-masseter, PPT-TA	Cross-sectional design; no subgroup analysis by gender/age; sleep disturbances not evaluated; single-center	High (cross-sectional design, single-center, no temporal validation)	No (AUC-ROC only)	No missing data strategy; no calibration; cross-sectional design limits prognostic interpretation
Jha *et al*. [26] (2022)	Systematic review of AI in TMD diagnosis	Multiple supervised algorithms (SVM, ANN, CNN, RF)	84–99.9% diagnostic accuracy; uniformly high risk of bias	Diagnostic classification only; no prognostic studies identified in review	N/A (systematic review)	N/A	N/A (systematic review)
Farook *et al*. [27] (2021)	Systematic review of ML in dental/OFP	Various ML methods	Confirms concentration on diagnostic tasks; outcome prediction is largely absent	Heterogeneous inclusion criteria; no meta-analysis feasible	N/A (systematic review)	N/A	N/A (systematic review)
Bair *et al*. [48] (2013)	OPPERA cohort; 202 baseline risk factors	LASSO regression, random forest	Comorbid pain conditions, psychosocial factors, and pain sensitivity as top predictors	Prediction of incident TMD (not chronic outcomes); internal validation only	High (internal validation only; prediction of incident TMD, not chronic outcomes)	No	No penalization reported; no calibration; internal validation only
Lötsch & Ultsch (2018) [57]	Narrative review of ML in pain research	Conceptual review of ML applications	ML can identify pain phenotypes and complex data patterns	Non-TMD-specific; illustrative rather than empirical validation	N/A (narrative review)	N/A	N/A (narrative review)
Bair *et al*. [60] (2016)	OPPERA cohort; cluster analysis	Supervised cluster analysis (k-means + discriminant function)	Three biopsychosocial subgroups (adaptive, pain-sensitive, and global symptoms) were identified and shown to predict TMD outcomes	Cross-sectional cluster assignment; no external validation	High (cross-sectional cluster assignment, no external validation)	No	Cluster assignment without temporal validation; no calibration
Al Turkestani *et al*. [61] (2024)	106 TMJ OA subjects (74 followed up after 2–3 years); prospective longitudinal; multimodal data (clinical, imaging radiomics, serum/saliva biomarkers)	EHPN framework integrating 18 feature selection and ML methods with SHAP interpretation	Accuracy 0.87, AUC 0.72, F1 0.82; personalized predictors: headache, back pain, sleep quality, condylar radiomics, joint space, mouth opening, and serum/saliva biomarkers	Small sample size (n = 74 follow-up); complex multimodal data may limit clinical accessibility; single-center prospective study	High (small sample n = 74 follow-up, single-center)	No (AUC-ROC only)	Small follow-up (n = 74) with high predictor-to-sample ratio; no calibration reported

*TMD: temporomandibular disorders; AI: artificial intelligence; ML: machine learning; ADD: anterior disc displacement; ANN: artificial neural network; AUC: area under the curve; CNN: convolutional neural network; CROM: cervical range of motion; DNN: deep neural network; DT: decision tree; EHPN: ensemble via hierarchical predictions through nested cross-validation; GBDT: gradient boosting decision tree; HADS: hospital anxiety and depression scale; LASSO: least absolute shrinkage and selection operator; LR: logistic regression; MARS: multivariate adaptive regression spline; MLP: multi-layer perceptron; MMO: maximum mouth opening; OBC: oral behaviors checklist; OHIP-14: oral health impact profile; OPPERA: orofacial pain prospective evaluation and risk assessment; OA: osteoarthritis; PPT: pressure pain threshold; RF: random forest; AUC-ROC: Area under the curve–receiver-operating characteristic; SHAP: SHapley Additive exPlanations; SVM: support vector machine; TA: temporalis anterior; VAS: visual analog scale; XGBoost: extreme gradient boosting; N/A: not applicable; PROBAST: Prediction model Risk Of Bias Assessment Tool; TMJ: temporomandibular joint; DC/TMD: Diagnostic Criteria for Temporomandibular Disorders; MRI: magnetic resonance imaging; OFP: orofacial pain; LightGBM: Light Gradient Boosting Machine.*

Although nearly all studies in Table 2 were rated as having a high risk of bias, the evidence base is not entirely at a standstill. Certain studies represent partial steps toward methodological maturity: Al Turkestani *et al*. [61] incorporated prospective longitudinal follow-up with multimodal data integration, while the OPPERA-based analyses [16, 48, 60] demonstrated the prognostic value of large cohort, multidimensional data collection approaches. However, no single existing study satisfies all criteria required for a well-developed prognostic AI model in TMD/OFP, namely, a prospective design, multimodal biopsychosocial data, clearly defined longitudinal outcome, external validation, calibration assessment, model explainability, and formal bias evaluation. The field is therefore best described as being at an early foundational stage, with individual studies contributing important building blocks, although none yet provides a complete template for future work.

### 4.4 Imaging-centered AI models: limited prognostic value

Imaging-based AI models, which have typically been developed to analyze panoramic radiographs or cone-beam computed tomography images for TMJ osteoarthritis diagnosis (DC/TMD: defined degenerative joint disease), are among the most technically developed areas of AI research in the TMD field [26, 46]. Deep learning models have demonstrated strong performance in detecting structural abnormalities in the TMJ [64, 65, 66]. Nevertheless, structural abnormalities in the TMJ have been reported to have a weak or even negligible relationship with patients’ clinical signs and symptoms, including pain intensity, functional disability, and long-term prognosis [47]. Moreover, current imaging-based models do not incorporate functional indicators such as jaw movement dynamics or masticatory muscle activity, nor do they integrate neurological biomarkers such as quantitative sensory testing profiles or indices of central sensitization, which may carry greater prognostic relevance for pain persistence than static structural findings alone.

This limitation is clinically important. Over-reliance on imaging-based prognostic signals may perpetuate outdated biomedical thinking and lead to unnecessary interventions rather than informed, conservative management [5, 6], which can worsen patients’ conditions. Such an approach also neglects the importance of holistic care. More generally, prognostic models that prioritize structural imaging over psychosocial factors may produce inaccurate predictions in relation to chronic pain, which is inconsistent with the goals of patient-centered, conservative care. A further risk that deserves explicit attention is over-medicalization. When AI models are trained predominantly on imaging features, such as condylar morphology, disc position, or joint space measurements, without adequate weighting of psychosocial phenotypes, structural variations that may be incidental rather than pathological can be inappropriately flagged as clinically significant. For example, disc displacement, including DC/TMD disc displacement categories, has been reported in a substantial proportion of asymptomatic individuals [67], and mild condylar remodeling may reflect a physiological process rather than disease progression. An AI model that flags these findings as high risk, without considering the patient’s pain experience, psychological status, or functional impact, does not resolve a clinical issue; instead, it may create one. Such models may lead to unnecessary imaging referrals, invasive procedures, or patient anxiety, all of which are inconsistent with the conservative biopsychosocial approach supported by current evidence for the management of TMD [6, 7, 9, 52]. For prognostic modeling in TMD/OFP, structural data should therefore be contextualized as one input among many within the patient’s broader clinical and psychosocial profile, rather than being used as the dominant or sole basis for risk stratification.

## 5. Methodological limitations and risk of bias

Examination of the PROBAST assessment at the domain level reveals a consistent pattern. The Analysis domain is the most frequent source of high risk of bias across the studies reviewed. None of the included prognostic studies reported calibration plots or calibration-in-the-large statistics; all relied exclusively on discrimination metrics such as AUC or accuracy. Strategies for handling missing data were either absent or not described in the majority of studies, and techniques to reduce over-optimism, such as penalized regression or bootstrap-based shrinkage, were not employed in any of the included studies. This pattern is not unique to TMD/OFP; Wynants *et al*. [68] identified similar systematic weaknesses across 731 COVID-19 prediction models. The absence of calibration is particularly concerning in the prognostic setting, where the clinical task involves not only ranking patients by risk but also generating probability estimates that can meaningfully inform treatment planning and shared decision-making [17, 28].

A related but distinct threat is data leakage, in which information that would not be available at the time of prediction is inadvertently incorporated during model training. In prognostic modeling, this risk is particularly relevant when repeated-measures data or electronic health records are used, as variables recorded after the index date—including subsequent treatment decisions, follow-up imaging, or later symptom scores—may be mistakenly treated as baseline predictors. Similarly, cross-sectional studies that assign outcome labels based on data collected at the same time point as the predictors cannot be considered truly prognostic, even if the analysis is presented as predictive. Among the studies reviewed, explicit safeguards against data leakage, such as strict temporal splitting in which the training set includes only data collected before a defined cutoff and the test set includes data collected afterward, were not reported. Future prognostic studies in TMD/OFP should therefore: (ⅰ) clearly define the index date (*i.e.*, the point at which prediction is made); (ⅱ) restrict all predictor variables to information available at or before that date; and (ⅲ) use time-based rather than random data splits for validation [17, 28, 29]. Table 3 (Ref. [28, 29, 30, 45]) summarizes the recurring methodological shortcomings identified across the reviewed studies alongside the corresponding ideal clinical standards for AI-based prognostic modeling in TMD/OFP.

**Table 3.** Existing AI-based prognostic studies versus ideal clinical standards in TMD/OFP.

Domain	Existing AI Studies in TMD/OFP	Ideal Clinical Standards
Study Design	Cross-sectional; diagnostic classifiers retrofitted as prognostic	Prospective longitudinal cohort design; prognosis-first design with predefined follow-up periods
Outcome Definition	Surrogate or imaging endpoints; inconsistent thresholds	Clinically meaningful outcomes: pain persistence, functional recovery, and treatment response
Sample Size	Small, single-center cohorts (<200 patients typically)	Adequately powered multicenter datasets; sample size calculation reported
Data Domains	Predominantly imaging or clinical variables; psychosocial factors are underrepresented	Integrated biopsychosocial data: clinical variables, psychosocial factors, Patient-reported outcome measures (PROMs), imaging (contextualized)
Validation	Internal cross-validation only; external validation absent	Robust external validation using independent, geographically diverse cohorts
Transparency	Black-box architectures; limited reporting of missing data or calibration	TRIPOD + AI–compliant reporting; explainable AI approaches; calibration assessed
Clinical Utility	No actionable risk stratification; outputs not mapped to decision points	Clinician-interpretable risk categories aligned with stepped-care pathways
Ethics/Equity	Bias assessment absent; subgroup performance unreported	Explicit fairness evaluation; subgroup performance; bias mitigation strategies implemented

*Ideal clinical standards are derived from established prognostic research frameworks, including TRIPOD + AI [29], PROBAST [28], and the PROGRESS prognosis research strategy [30, 45]. TRIPOD: Transparent Reporting of a Multivariable Prediction Model for Individual Prognosis or Diagnosis; TMD: temporomandibular disorders; AI: artificial intelligence; OFP: orofacial pain.*

### 5.1 Study design misalignment

A fundamental limitation across the existing literature is the mismatch between study design and prognostic objectives. Prognostic modeling requires longitudinal data with clearly defined follow-up periods and outcome measures [4, 38]. Many AI studies labeled “predictive” in TMD and OFP have relied on cross-sectional datasets or short-term follow-up, making true prognostic inference methodologically invalid [30]. Consequently, these studies often produce models that predict contemporaneous states rather than future outcomes, a distinction that is frequently obscured in reporting [17, 56]. Given that most reviewed studies lacked a clear temporal separation between input variables and outcomes, the risk of data leakage, as discussed in Section 5, cannot be excluded, further undermining the validity of their prognostic claims.

### 5.2 Outcome definition and clinical relevance

Existing studies frequently utilized highly variable and poorly justified prognostic endpoints of limited clinical relevance, such as changes in pain thresholds or reductions in imaging abnormalities [14, 39]. These endpoints often bear little relationship to clinically meaningful outcomes, such as overall treatment response, degree of clinical recovery, and risk of persistent clinically significant pain states [6, 7]. Thus, such studies may yield statistically significant prediction models that have limited bearing on clinical decision-making in practice [17].

### 5.3 Sample size, data quality, and overfitting

Small sample sizes are a recurring limitation in the reviewed studies. ML models, particularly high-dimensional models, are prone to overfitting when trained on limited data [56]. Overfitted models may perform well internally but degrade substantially when applied to new patient populations. Compounding this limitation, many studies failed to report details on data preprocessing, missing data handling, or class imbalance, all of which can substantially influence model performance and reproducibility [30, 69]. These reporting gaps have also been highlighted in a commentary on the reporting of AI prediction models, which identified heterogeneous design and limited reproducibility as recurring concerns across clinical domains [70]. These issues mirror the broader pattern identified by Wynants *et al*. [68] across COVID-19 prediction models.

### 5.4 Absence of external validation

The process of validating models on independent datasets is essential to the development of any robust prognostic model [34, 71]. Studies of AI in OFP rarely include external validation, and validation methods beyond cross-validation are seldom used [34, 71]. External validation is particularly important in TMD/OFP due to the large number of relevant variables, including diverse psychosocial risk factors, variable levels of access to healthcare, and differing clinical practice patterns, all of which may affect the performance of a model developed under different conditions. Without external validation, the generalizability of these models remains uncertain [70]. The concept of domain shift is especially relevant to OFP management. Most AI models were developed in tertiary orofacial pain centers, where patients tend to present with more complex, chronic, and treatment-resistant conditions. When these models are applied in primary care or general dental settings, where patients often present with earlier-stage disease and different psychosocial profiles and comorbidities, they may systematically overestimate chronicity risk or misclassify treatment response. This problem is further compounded by differences in clinical assessment protocols (*e.g.*, extent of DC/TMD Axis II evaluation), referral thresholds, socioeconomic context, and cultural variation in pain expression across geographic regions [34, 71]. Domain shift is therefore not merely a statistical issue but also a clinical concern, affecting equitable and appropriate pain management. This underscores the necessity of validating prognostic models across geographically and sociodemographically diverse populations.

### 5.5 Reporting quality, model transparency, and explainability

Complex and opaque algorithms may pose barriers to practical clinical use, even though deep learning methods can model more complex, nonlinear patterns than simpler algorithms. Such models are generally less interpretable, and clinicians must reconcile this limited interpretability with the need for clinical and social trust, participation in shared decision-making, and adherence to ethical principles [18, 55]. In pain medicine, where clinical estimates of prognosis routinely inform patient education, counseling, and communication of post-surgical expectations, a TMD/OFP model that produces a prognostic estimate should also provide a corresponding clinical explanation. Complex models may give the impression of interpretability that exceeds their actual transparency and may therefore fall short of the clinical utility requirements in practice [55].

As noted above, none of the prognostic studies examined reported model calibration, relying exclusively on discrimination metrics such as AUC and accuracy (Table 2). In prognostic modeling, calibration—the alignment between predicted probabilities and actual outcome frequencies—is often more clinically relevant than discrimination, as it directly affects the reliability of personalized risk estimates communicated to patients and healthcare professionals [28, 29]. When a prognostic model reports strong discrimination without calibration, there is no assurance that a predicted probability (*e.g.*, 70%) corresponds to an actual observed risk near that value, a gap that could lead to inappropriate treatment escalation or false reassurance if acted upon clinically [14, 17].

### 5.6 Bias and equity

Bias amplification represents a further, often overlooked risk. When AI prognostic models are trained on datasets that inadequately represent specific demographic or psychosocial subgroups, they may generate systematic errors in risk assessment, potentially exacerbating existing inequalities in pain management [33, 35]. This issue is not merely theoretical. Obermeyer *et al.* [35] demonstrated that a widely used commercial healthcare algorithm systematically underestimated the health needs of Black patients because it relied on healthcare cost as a proxy for illness severity, a measure influenced by access to care rather than true clinical need. This finding is particularly relevant for TMD and OFP, where sex-based prevalence differences, psychosocial factors linked to socioeconomic status, and differential access to specialized care can similarly distort model training and subsequent prognostic predictions [33]. If prognostic AI models for TMD/OFP are developed without attention to these factors, they may inadvertently perpetuate or worsen disparities. Few existing studies assess fairness, subgroup performance, or bias mitigation strategies. A similar concern applies to DC/TMD Axis II measures: high somatic symptom burden scores may reflect true symptom severity but can also be influenced by reporting style, cultural context, or comorbidities. If such scores are used uncritically in model training, the resulting model may treat somatic symptom burden as inherently prognostic of chronicity, thereby reinforcing assumptions rather than empirically testing them. Reliance on such outputs risks generating self-fulfilling prognostic predictions. Psychosocial variables should therefore be interpreted within validated clinical frameworks, and bias auditing should be a standard component of model development.

## 6. What is missing and how can it be fixed?

As previously discussed, the literature suggests a growing interest in AI applications within TMD and OFP research. However, none of the existing studies have addressed the most clinically meaningful prognostic questions for healthcare providers and patients. This section outlines the identified gaps and proposes methodological priorities for future research.

### 6.1 The prognosis question nobody is asking

All current AI studies were designed around diagnostic questions, with prognostic claims made only retrospectively. As discussed in Section 5.1, this design misalignment limits the prognostic validity of existing models. To close this gap, future studies should adopt a prognosis-first design, beginning with clinically relevant prognostic questions, such as chronicity risk, likelihood of success with conservative therapy, and risk of symptom recurrence, and should define cohort selection criteria, follow-up duration, outcome definitions, and modeling approaches accordingly [4, 38, 71]. Standardized data collection based on validated assessment tools, such as DC/TMD Axis II and patient-reported outcome measures (PROMs), would facilitate cross-study comparison and multicenter collaboration.

### 6.2 The cost of ignoring time

As highlighted in Section 5.1, the current literature is predominantly based on cross-sectional data. Consequently, the temporal characteristics of pain trajectories are not captured. Temporal modeling approaches discussed in Section 3.3, including survival analysis-augmented ML [50], mixed-effects modeling combined with ML, and RNN architectures [51], have not yet been applied in the context of TMD/OFP. However, evidence from other pain and medical fields suggests that these modeling approaches could enable a more nuanced understanding of the temporal characteristics of pain, potentially identifying key transition points amenable to early intervention.

### 6.3 Why are psychosocial data still an afterthought?

As discussed in Section 4.4, current AI models continue to underrepresent psychosocial variables. Without such data in training datasets, algorithms learn to depend on imaging and clinical features. This creates a self-reinforcing cycle whereby the absence of psychosocial data today makes their inclusion in future studies less likely [9, 10, 11, 12, 16, 63]. Imaging data constitute only one component of a comprehensive prediction model, and biopsychosocial integration is essential for meaningful prognostic research. There is also a technological dimension to this problem. ML algorithms tend to assign greater weight to clinical and imaging variables, not because these are inherently stronger predictors, but because they are collected more consistently, carry less measurement noise, and are represented in standardized numeric formats. Psychosocial data, by contrast, are typically gathered through self-report instruments that can exhibit floor and ceiling effects, vary across cultures, and appear less informative to algorithms optimized for structured inputs. Overall, these considerations, together with evidence repeatedly demonstrating psychosocial factors as important prognostic markers [9, 10, 16], underscore the need to break this cycle by purposefully incorporating validated psychosocial assessment tools into future model development.

### 6.4 Models that have never left the lab

The near-universal absence of external validation, as discussed in Section 5.4, means that no current model can be safely assumed to generalize beyond its development cohort. Multicenter datasets, federated learning [72, 73], and transparent reporting frameworks such as TRIPOD + AI [29] can improve generalizability while protecting data privacy. Federated learning is particularly promising, as it enables multiple institutions to jointly train a shared model without exchanging raw patient data. Prospective validation studies, although resource-intensive, carry particular value for establishing clinical credibility and informing regulatory pathways [34, 71].

### 6.5 When predictions do not lead to decisions

Many AI models produce probabilistic outputs, which can be difficult to interpret in clinical settings. Prognostic estimates that do not map onto actionable thresholds, such as low-, moderate-, or high-risk categories, offer limited utility to practitioners managing complex pain conditions [6, 7]. Explainability should be prioritized alongside predictive performance. To ensure model outputs align with clinical reasoning and workflow, hybrid modeling approaches that balance interpretability and accuracy should be adopted, incorporating clinician-in-the-loop development processes [18, 55]. In practical terms, a prognostic model for TMD/OFP should not merely yield a probability of chronicity but should translate that estimate into recommendations that clinicians can interpret and that align with established care pathways. For example, it should flag when a patient’s psychosocial risk profile suggests a low chance of responding to conservative splint therapy alone, prompting early consideration of multidisciplinary interventions, including cognitive-behavioral therapy. Importantly, the probability thresholds that trigger transitions between care levels (*e.g.*, from conservative management to multidisciplinary intervention) cannot be prescribed a priori; they must be calibrated through prospective studies that map model-derived risk strata onto observed treatment outcomes across diverse clinical populations. Explainability methods, such as SHAP and LIME, can identify which variables drive individual predictions, but their values are model-specific and should not be interpreted as universal clinical cutoffs. Close collaboration among clinicians, data scientists, and behavioral researchers is essential to ensure models highlight modifiable risk factors, such as stress, sleep disturbance, and parafunctional behaviors, enabling targeted interventions. Fig. 2 illustrates the proposed end-to-end prognostic AI pipeline for TMD/OFP, spanning from multimodal biopsychosocial data collection to prospective outcome evaluation.

**Fig. 2.** Proposed prognostic AI pipeline for OFP and TMD. The pipeline spans six stages: (1) multimodal baseline data collection, (2) longitudinal data integration, (3) AI/ML model development with explainability and validation, (4) prognostic outputs, (5) clinician-in-the-loop decision support, and (6) prospective outcome evaluation with feedback for model refinement. Cross-cutting requirements include TRIPOD + AI, PROBAST, calibration assessment, fairness metrics, and stakeholder engagement. (Figure constructed based on the frameworks and evidence discussed in this review using Microsoft® PowerPoint for Mac, V. 16.107). AI: artificial intelligence; ML: machine learning; DC/TMD: diagnostic criteria for temporomandibular disorders; PHQ-9: patient health questionnaire-9; GAD-7: generalized anxiety disorder-7; TMJ: temporomandibular joint; MRI: magnetic resonance imaging; CBCT: cone-beam computed tomography; NRS: numeric rating scale; VAS: visual analog scale; QoL: quality of life; OHIP: oral health impact profile; SF-36: 36-item Short Form Health Survey; RNNs: recurrent neural networks; SHAP: SHapley additive explanations; LIME: local interpretable model-agnostic explanations; CV: cross-validation; TRIPOD: Transparent Reporting of a Multivariable Prediction Model for Individual Prognosis or Diagnosis; PROBAST: Prediction Model Risk of Bias Assessment Tool.

To translate prediction into decision support, it is necessary to define what constitutes an actionable prognostic output in TMD/OFP care. A binary classification, such as “high risk of chronicity”, or a probability score in isolation, provides limited guidance to the treating clinician. A more useful output should not only estimate risk but also identify which modifiable factors contribute most to that risk for an individual patient. For instance, if a model determines that a patient’s elevated risk of persistent pain is driven primarily by catastrophizing, sleep disturbance, and parafunctional habits rather than by structural joint findings, this information could guide referral toward cognitive-behavioral therapy, sleep hygiene counseling, or behavioral modification, rather than further imaging or surgical consultation. In this way, the model output becomes directly linked to a specific, individualized management pathway. Achieving this level of clinical utility requires that prognostic models incorporate explainability methods [53, 55] and that outputs are developed collaboratively with clinicians, ensuring the information provided is both statistically valid and interpretable within existing care workflows [39, 74]. Without this step, even a well-calibrated and externally validated model is unlikely to meaningfully impact clinical practice.

### 6.6 Equity and accountability: unfinished business

As discussed in Section 5.6, bias assessment is largely absent from existing studies. Given the well-established sex differences and psychosocial disparities in TMD/OFP prevalence and outcomes [4, 16], explicit assessment of subgroup performance, fairness metrics, and bias mitigation strategies should be regarded as a minimum standard for future research [33, 35]. Table 4 summarizes the identified research gaps, along with suggested actions and their corresponding priority levels. In addition, Fig. 3 presents a phased research roadmap, illustrating the progression of AI-driven prognostic modeling from initial data collection to eventual clinical application.

**Table 4.** Research gaps and recommended actions for AI-based prognostic modeling in TMD/OFP.

Research Gap	Current Status	Recommended Action	Priority Level
Prognosis-first study design	Nearly absent; diagnostic classifiers repurposed	Design studies around explicit prognostic questions with longitudinal follow-up data	Critical
Psychosocial domain integration	Underrepresented in most AI models	Include validated psychosocial instruments (DC/TMD Axis II, PHQ-9, GAD-7, PSQI) as core predictor variables	Critical
Longitudinal modeling	Most studies use cross-sectional data	Adopt RNNs, or mixed-effects ML approaches	High
External validation	Absent in nearly all existing studies	Establish multicenter collaborations; explore federated learning for data sharing	Critical
Clinically actionable outputs	Probabilistic outputs without decision thresholds	Map model outputs to risk categories aligned with stepped-care clinical pathways	High
Ethical and equity evaluation	Rarely or never assessed	Mandate subgroup performance reporting, bias auditing, and fairness metrics	High
Reporting standards	Inconsistent; TRIPOD + AI rarely followed	Adopt TRIPOD + AI and PROBAST as minimum reporting requirements for all prognostic AI studies	Critical

*AI: artificial intelligence; DC/TMD: Diagnostic Criteria for Temporomandibular Disorders; PHQ-9: Patient Health Questionnaire-9; GAD-7: Generalized Anxiety Disorder-7; PSQI: Pittsburgh Sleep Quality Index; RNN: recurrent neural network; ML: machine learning; TRIPOD: Transparent Reporting of a Multivariable Prediction Model for Individual Prognosis or Diagnosis; PROBAST: Prediction Model Risk of Bias Assessment Tool.*

**Fig. 3.** Proposed research roadmap for AI-based prognostic modeling in TMD/OFP. This figure presents a staged research roadmap spanning three phases: (Phase 1) Foundation, establishing standardized longitudinal data collection with biopsychosocial integration and validated outcome measures; (Phase 2) Model Development, applying trajectory-based ML methods, integrating explainability, and conducting rigorous internal validation; and (Phase 3) Translation, involving multicenter external validation, federated learning implementation, clinical decision support integration, and prospective outcome evaluation. Black arrows indicate progression from one phase to the next. Each phase includes recommended methodological standards (TRIPOD + AI, PROBAST), ethical safeguards, and milestones for assessing readiness to proceed (shown using colored arrows). (Figure constructed based on the frameworks and evidence discussed in this review using Microsoft® PowerPoint for Mac, V. 16.107). DC/TMD: Diagnostic Criteria for Temporomandibular Disorders; ML: machine learning; PROMs: patient-reported outcome measures; RNNs: recurrent neural networks; SHAP: SHapley Additive exPlanations; LIME: Local Interpretable Model-Agnostic Explanations; k-fold CV: k-fold cross-validation; TRIPOD + AI: Transparent Reporting of a Multivariable Prediction Model for Individual Prognosis or Diagnosis plus Artificial Intelligence; EHR: electronic health record; PROBAST: Prediction Model Risk of Bias Assessment Tool.

## 7. From algorithm to clinic: ethics, translation, and practical realities

Even if the methodological limitations outlined above were addressed, significant challenges would remain in moving prognostic AI tools from research to clinical use. This section addresses the ethical obligations, translational barriers, and practical considerations that will determine whether these tools ultimately reach clinical practice.

### 7.1 Communicating uncertainty without doing harm

Unlike diagnostic classifications, the value of a prognostic prediction lies primarily in its impact on clinical management. The uncertainty inherent in prognostic predictions can affect the management of chronic pain conditions, where patients’ beliefs and expectations play a crucial role in their clinical outcomes. Providing a patient with a probabilistic risk estimate for developing chronic pain can influence their symptom experience, coping behavior, and adherence to treatment recommendations [10, 11]. A binary prediction indicating that a patient is at high risk of developing chronic pain can reinforce maladaptive beliefs and catastrophizing in psychologically vulnerable patients [9, 16].

Communicating uncertainty in prognostic prediction can therefore complicate chronic pain management. Development of AI-based tools to guide communication of risk estimates could support shared decision-making with patients. As with diagnostic predictions, prognostic AI tools must communicate predictions as risk estimates with confidence ranges rather than as definitive outcomes, in order to avoid undermining the patient’s ability to manage their condition and focus on modifiable risk factors such as stress management, sleep hygiene, and adherence to conservative therapy [10, 11].

### 7.2 Explainability as an ethical necessity in prognostic AI

Explainability is not merely a technical preference but an ethical necessity in prognostic AI applications. Clinicians must understand which variables contribute most strongly to prognostic estimates to justify clinical decisions and identify potentially modifiable risk factors [18, 55]. Methods such as LIME [53] and SHAP [54] can substantially enhance interpretability without sacrificing predictive performance, enabling responsible use in clinical contexts.

### 7.3 Whose pain counts? Bias and fairness in prognostic AI

Ethical model development requires explicit evaluation of subgroup performance, transparency regarding data sources, and the application of fairness metrics [33, 35]. A model that exacerbates disparities in pain management, either by undertreating higher-risk subgroups or overtreating lower-risk subgroups, is ethically unacceptable. As Char *et al*. [75] argued, general ethical considerations for the use of ML in healthcare—such as informed consent, data privacy, algorithmic transparency, accountability for clinical decisions, and potential for bias—are broadly applicable but carry particular importance in pain management, given the central role of subjective experience, individual preferences, and social determinants of health [75].

### 7.4 Keeping the clinician in the loop, and making it work in practice (clinical responsibility)

AI-based prognostic models should function as decision-support tools, not autonomous decision-makers. The treating clinician should retain full clinical responsibility and use model predictions in combination with their own assessment, clinical judgment, patient preferences, and clinical context [6, 7]. Price *et al*. [76] have highlighted the need for legal frameworks addressing liability when AI tools inform clinical decisions [76]. Such tools must always be interpreted as decision aids rather than autonomous systems [74].

For practitioners managing TMD and chronic OFP, AI-based prognostic modeling offers the potential to improve risk stratification, patient communication, and treatment planning (Table 5). Even after an exact DC/TMD diagnosis, patients with the same diagnosis are often managed differently across clinics and over time, and the condition can progress at markedly different rates [1, 5]. Prognostic models integrating clinical, psychosocial, and behavioral data could help identify patients at higher risk of persistent pain or functional deterioration early in the care pathway, enabling tailored management, including closer monitoring, earlier multidisciplinary involvement, or targeted psychosocial interventions for high-risk individuals, while avoiding overtreatment in lower-risk patients [6, 7].

**Table 5.** Potential clinical applications of AI-based prognostic models in TMD/OFP.

Clinical Application	Description	Practical Example	Prerequisite Conditions
Early risk stratification	Identify patients at high risk of chronic pain or disability at initial presentation	New TMD patient flagged as high-risk based on psychosocial profile and symptom patterns; triaged for early multidisciplinary assessment	Validated prognostic model with external validation; integrated biopsychosocial data collection
Treatment response prediction	Estimate the likelihood of response to specific interventions (*e.g.*, splint therapy, CBT, physical therapy)	Model predicts low probability of response to splint therapy alone; clinician discusses combined approach with patient	Longitudinal treatment outcome data; model trained on multiple intervention types
Shared decision-making support	Provide probabilistic risk estimates to facilitate informed patient–clinician discussions	Clinician presents risk range: “Based on your profile, there is a 30–50% chance of significant improvement with conservative care over 6 months.”	Explainable model outputs; clinician training in AI-assisted communication
Monitoring and follow-up planning	Guide intensity and frequency of follow-up based on predicted trajectory	Low-risk patients are scheduled for standard 3-month follow-up; high-risk patients receive monthly check-ins and psychosocial support	Dynamic risk updating capability; integration of EHR-based data capture
Resource allocation	Direct limited multidisciplinary resources toward patients most likely to benefit	Priority referral to pain psychology or physical therapy for patients with predicted psychosocial-driven chronicity	Validated risk thresholds; institutional workflow integration

*CBT: cognitive-behavioral therapy; TMD: temporomandibular disorders; EHR: electronic health record; AI: Artificial Intelligence.*

These strategies must be implemented without imposing undue burden on practitioners [75, 76]. Ideally, tools would be integrated within electronic health record systems or embedded in existing workflows where data are already collected during a DC/TMD Axis I and II evaluation. Clinician involvement in tool design is essential to ensure usability, relevance, and trust. AI outputs should always be interpreted within the context of a comprehensive clinical assessment, and the resulting recommendations should not be considered mandatory or definitive recommendations. Natural language processing (NLP) offers a potential approach to incorporating psychosocial information into prognostic models. Clinical notes in electronic health records frequently contain rich narrative accounts of patients’ pain experiences, coping strategies, sleep patterns, and social contexts, which are routinely recorded by clinicians but seldom used for model training. NLP methods could extract and structure this narrative data, potentially providing a more clinically meaningful representation of a patient’s psychosocial profile than questionnaire scores alone. Although NLP applications in TMD/OFP have yet to be explored, their increasing use in other chronic pain domains suggests that this may represent a promising direction for future research [77].

## 8. Strengths and limitations

This review has several strengths. It is among the first to critically appraise prognostic AI in TMD/OFP as a field distinct from diagnostic AI. The included literature was evaluated against established frameworks (TRIPOD + AI, PROBAST, PROGRESS), providing a structured benchmark for methodological quality. The review integrates AI/ML methodology, clinical evidence, ethical considerations, and translational barriers within a single, cohesive analysis. It also proposes an actionable prognosis-first research roadmap (Section 6, Table 4, Fig. 3) and a prognostic AI pipeline (Fig. 2), and explicitly advocates for the integration of psychosocial and behavioral data alongside clinical and imaging variables, reflecting the biopsychosocial model that existing AI studies have largely neglected.

Several limitations should be acknowledged. As a conceptual review, no formal Preferred Reporting Items for Systematic Reviews and Meta-Analyses (PRISMA) flow diagram or quantitative meta-analysis was performed; risk of bias was appraised narratively using PROBAST rather than as a formal scored synthesis, and the scope of inclusion reflects the author’s judgment. The search covered four databases (PubMed/MEDLINE, Web of Science, IEEE Xplore, Google Scholar). Scopus was not accessible during revision, which may have reduced the capture of engineering-focused ML publications, despite mitigation through IEEE Xplore and Web of Science. Furthermore, only English-language publications were included, which may limit bibliographic completeness. The AI/ML literature is evolving rapidly, and the observations here reflect the state of the field at the time of writing. Because most AI work in TMD/OFP remains cross-sectional and diagnosis-oriented, several prognostic recommendations draw on evidence from adjacent pain domains (*e.g.*, low back pain, chronic widespread pain), whose transferability to TMD/OFP has not been empirically validated. Finally, this review did not incorporate patient or clinician stakeholder input. These limitations constrain the scope of the review’s claims but do not diminish its central argument: the field requires prognosis-first study designs, longitudinal data, explainable models, and adherence to modern reporting standards.

## 9. Future directions

Advancing AI-driven prognostic modeling for TMD and chronic OFP requires coordinated efforts across several strategic domains. First, multicenter prospective longitudinal cohorts should be established, building on the OPPERA model by gathering standardized biopsychosocial data, including DC/TMD Axis I and II evaluations, patient-reported outcome measures, and psychosocial tools (PHQ-9 (Patient Health Questionnaire-9), GAD-7 (Generalized Anxiety Disorder-7), PSQI (Pittsburgh Sleep Quality Index)) from diverse geographical and demographic populations. Such datasets are essential for developing generalizable and externally valid AI prognostic models. Second, methodological development should prioritize trajectory-focused and dynamic modeling techniques. Survival analysis-augmented ML and RNNs are well-suited to forecasting the nonlinear pain trajectories associated with TMD and chronic OFP. This prognostic capability can help identify key transition events, including the progression from acute to chronic pain. Moreover, by incorporating real-time symptom changes, digital health technologies—including wearable sensors and ecological momentary assessment (repeated real-time sampling of patient-reported outcomes in everyday settings)—could enhance model accuracy. Third, federated learning and privacy-preserving methods provide a mechanism for cooperative model training across institutions without the need for centralized data exchange, thereby facilitating external validation while protecting patient confidentiality and minimizing data fragmentation.

Fourth, employing clinician-in-the-loop design methods ensures that the cooperative creation of prognostic tools engages both patients and clinicians. This approach helps guarantee that the instruments reflect patient-centered objectives and clinical reasoning, accounting for the likelihood of functional recovery and modifiable risk factors. Fifth, the ethical framework must evolve in parallel with technical developments, including calibrated bias auditing techniques for pain management, clear reporting of subgroup performance stratified by sex, race, and socioeconomic status, and governance mechanisms that define clinical responsibility for AI-informed decisions. Sixth, clinical studies are needed to evaluate the impact of AI-assisted prognostic technologies on patient outcomes, clinical decision-making, and healthcare resource utilization.

Ultimately, even well-designed models will face a practical ceiling: without comprehensive, high-quality datasets that capture the full biopsychosocial spectrum of TMD/OFP, clinical deployment will remain premature. Until such data infrastructure is in place, these tools are best regarded as research instruments, with clinical translation dependent on the sustained, collaborative effort outlined above.

## 10. Conclusion

AI-based prognostic modeling for TMD and chronic OFP remains largely in the exploratory stage. The main barrier is conceptual rather than technological: the field has not yet adopted a prognosis-first approach, longitudinal data collection, or deliberate integration of the biopsychosocial factors that drive chronic pain trajectories. This review examined the types of outcomes predicted, data modalities utilized, modeling approaches adopted, validation strategies employed, and reporting quality across the existing literature, revealing consistent shortcomings in each of these domains.

The path forward is feasible and clinically relevant. Prioritizing longitudinal modeling, multidisciplinary biopsychosocial data integration, explainability, ethical monitoring, and adherence to TRIPOD + AI reporting standards can advance prognostic research beyond proof-of-concept toward tools that meaningfully support clinical decision-making. AI-based prognostic research in TMD/OFP should now prioritize methodological rigor and clinical relevance to ensure that emerging tools are scientifically sound and practically useful. Sustained investment in this research agenda may yield clinically meaningful advances in TMD/OFP prognosis and management.
